# The Prevalence of HPV Genotypes in Iranian Population: An Update

**DOI:** 10.30699/ijp.2019.90356.1861

**Published:** 2019

**Authors:** Mina Mobini Kesheh, Hossein Keyvani

**Affiliations:** 1 Student Research Committee, Department of Virology, School of Medicine, Iran University of Medical Science, Tehran, Iran; 2 Department of Virology, School of Medicine, Iran University of Medical Science, Tehran, Iran

**Keywords:** Human papillomavirus (HPV), Age distribution, Genotype distribution, Iran, INNO-LiPA HPV genotyping

## Abstract

**Background & Objective:**

*Human papillomavirus* (HPV) is the main cause of genital warts and some anogenital cancers in male and female subjects which is commonly transmitted by sexual contacts. The objective of this cross-sectional study was to examine the prevalence of HPV genotypes in 10,266 Iranian male and female population, according to their age.

**Methods:**

Samples were collected from the penile and anal sites of male subjects and the vagina and cervix of female subjects in a time period between 2011 and 2016. HPV DNA was detected in PCR using the MY09 and MY11 primers, and the INNO-LiPA assay was applied for HPV genotyping. To investigate the relevance of HPV infection and age, the samples were classified into 4 age groups (13-29, 30-44, 45-59, and 60-74).

**Results:**

Totally, the most common low risk HPV genotypes detected in the studied male and female subjects were HPV-6 (77.7% and 43.3%) and HPV-11 (13.7% and 11.4%), and more frequent high risk HPV genotypes were HPV-16 (5.5% and 16.6%) and HPV-52 (3.2% and 9.6%), respectively. High burden of the HPV infection was observed at ranges of 30 and 44 years (51.8%) with a peak at ranges between 30 and 32 years. No considerable statistically significant correlation was found between HPV infection and age (*P*=1).

**Conclusion:**

This study gave an epidemiological overview of circulating HPV genotypes in Iranian population to develop future vaccination policies, though the findings of prevalent HPV genotypes in female subjects were inconsistent with the previous studies reported in Iran.

## Introduction

More than 210 *Human papillomavirus* (HPV) genotypes have been detected so far. Adequate documents show that there is a relationship between HPV and some human cancers such as cervix cancer, head and neck carcinoma, penile cancer, oropharyngeal squamous cell carcinomas and, anal cancers (1, 2). According to the most recent meta-analysis study conducted on Iranian healthy female and female subjects with cervical cancer, the prevalence of HPV infection was 9.4% and 77.4%, respectively. The predominant genotype in both groups was HPV-16 (3, 4). Based on an estimated worldwide study published by the international agency for research on cancer (IARC), the HPV prevalence in female subjects with normal cytology in Africa, Latin America, Northern America, Asia, and Europe was 22.9%, 18.6%, 13.8%, 8.3%, and 6.6%, respectively and in a descending order (5). In less developed countries, cervix cancer is the second most common cancer (5) caused mostly by HPV-16 and -18 (6); however, cervical cancer is the 12^th^ most common cancer among Iranian female subjects (7). In male subjects, HPV-16 is the most frequent HPV genotype detected in penile cancer, and HPV-6 and -11 are often seen in genital warts (8). Although few studies have been carried out on HPV genotype distribution in Iranian male subjects, a recent study reported the 55.7% prevalence of HPV with HPV-6 as the most frequent genotype (9). HPV is an important agent in sexually transmitted diseases; screening and HPV genotyping tests, especially in low and middle income countries where HPV vaccination programs are not established, may help to improve the public health and prevent the spread of HPV infection (10). Even though Gardasil 4-valent is available in Iran, no national program exists to administer the vaccine to girls and boys.

The main objective of this study was to evaluate the distribution of HPV genotypes among Iranian males and females and provide a perspective of the dominant genotypes in the country. Although different studies on female or male samples have been accomplished in various provinces of Iran, as far the researchers investigated, this study is the first survey in Iran with a large sample size and participation of both male and female subjects. The findings might be helpful not only for setting future investigations and an agenda for public health, but also for vaccine development and national vaccination planning. 

## Materials and Methods

Procedure

In this cross-sectional study, a total of 8,351 (81.3%) females and 1,915 (18.7%) males from different provinces of Iran who were referred to Keyvan Laboratory of Tehran for HPV DNA testing and genotyping between April 2011 and April 2016 were enrolled. Almost all subjects were sexually active with an age range from 13 to 74 years old. The HPV DNA testing and genotyping were ordered by clinicians. Male samples were collected from anal and penile biopsies and female samples from the vagina and cervix through the use of either Dacron swabs or brush. 

For DNA extraction, QIAamp DNA Mini kit (Qiagen GmbH, Germany) was utilized in accordance with the manufacturer’s directions and the concentration of extracted DNA was evaluated by spectrophotometry in 260 nm. Genomic DNA of the samples was used in PCR using the MY09 and MY11 primers. DNA quality in samples was verified using beta-globin PCR assay.

Detection of HPV DNA and genotypes were carried out using INNO-LiPA assay (Fujirebio Europe N. V., Belgium). The INNO-LiPA HPV genotyping is based on the principle of reverse hybridization. Briefly, a part of L1 region called SPF10 of HPV genome was extended by specific primers; then, the biotinylated amplimers were denatured and hybridized with specific oligonucleotide probes. Furthermore, a primer pair of the human HLA-DPB1 gene was added to confirm the quality and extraction of DNA. Next, streptavidin-conjugated alkaline phosphatase was added. After incubation with BCIP/NBT chromogen, the results were interpreted visually.

Statistical analysis

IBM SPSS statistics version 22 (11) was used for statistical analysis as follows: 95% CI for prevalence estimations and the relation of HPV infection with age was performed using the Kruskal-Wallis test. A P-value less than 0.05 was considered to be statistically significant.

## Results

**Figure 1:**
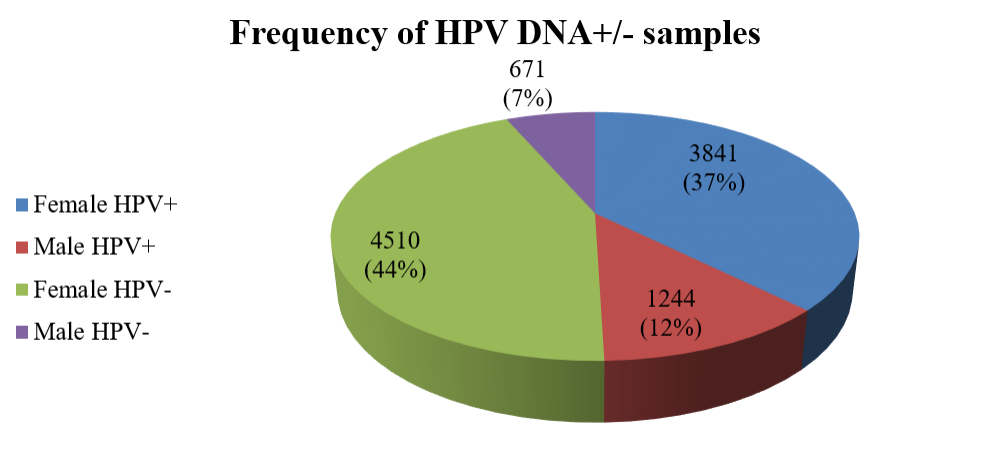
The percentage of HPV prevalence in the population (n=10266) of this study. HPV DNA was not detected in 44.0% and 6.5% of females and males, respectively; however, 37.4% of female subjects and 12.1% of male subjects were positive for HPV DNA

INNO LiPA HPV genotyping determined 32 HPV genotypes; HPV-16, -18, -31, -33, -35, -39, -45, -51, -52, -56, -58, -59, and -68 as high risk HPV genotypes (HR HPV), HPV-26, -53, -66, -70, -73, and -82 as possible high risk HPV genotypes (pHR HPV), and HPV-6, -11, -40, -42, -43, -44, -54, -61, -62, -67, -81, -83, and -89 as low risk HPV genotypes (LR HPV). This HPV classification was based on IARC Monographs (12). During the study period, the total HPV prevalence was 49.5%; hence, among 10,266 participants, 49.5% (n=5085) were HPV DNA positive and 50.5% (n=5181) were negative. Figure (1) displays the overall HPV prevalence according to sex. HPV DNA was not detected in 44.0% (n=4510) of females and 6.5% (n=671) of males while HPV was genotyped in 37.4% (n=3841) of female subjects and 12.1% (n=1244) of male subjects.

Totally, among 5,085 samples from 31 Iranian provinces, four most common HPV genotypes were HPV-6, -11, -16, and -52 in male subjects and HPV-6, -16, -11, and -52/-53 (HPV-52 and HPV-53 were equal in frequencies) in female subjects, in a descending order. Among HR and pHR HPV genotypes, after HPV-52/-53 (9.6%), HR HPV-51 (8.0%) had the highest frequency in female subjects and also other genotypes with prevalence less than 8% included pHR HPV-66 (7.7%) and HR HPV-31 (7.0%). In male subjects, HR HPV-51 (2.8%) was the third most common HR HPV genotypes. The common HPV genotypes was observed in both single and mixed HPV infections and the genotypes which had high rates in the single HPV infection were also frequent in the mixed infections (Tables 1 and 2). Tehran accounted for the most HPV DNA positive patients (40.1%) followed by Gilan (16.1%) and Mazandaran (5.6%) (Table 3).

**Table1:** The prevalence of HPV genotypes as a single infection in Iranian female and male population

Sex					
**Male**		Female			
**%, 95% CI**	No	%, 95% CI	No	HPV Genotypes	
**66.5 (64.0-68.9)**	828	26.1 (24.8-27.6)	1004	6	**LR HPV**
**10.5 (8.8-12.1)**	131	5.2 (4.5-6.0)	201	11	
**1.4 (0.8-2.2)**	18	0.7 (0.4-0.9)	26	44	
**0.2 (0.0-0.6)**	3	1.1 (0.8-1.4)	42	54	
**0.2 (0.0-0.4)**	2	0.7 (0.4-0.9)	26	62	
**0 (0.0-0.0)**	0	0.5 (0.3-0.8)	20	89	
**0 (0.0-0.0)**	0	0.2 (0.1-0.4)	9	43	
**0 (0.0-0.0)**	0	0.2 (0.1-0.4)	9	40	
**0 (0.0-0.0)**	0	0.4 (0.3-0.7)	17	61	
**0 (0.0-0.0)**	0	0.2 (0.1-0.4)	9	67	
**0 (0.0-0.0)**	0	0.3 (0.1-0.4)	10	81	
**0 (0.0-0.0)**	0	0.2 (0.1-0.4)	8	83	
**0.2 (0.0-0.6)**	3	0.3 (0.1-0.4)	10	42	
**0.2 (0.0-0.6)**	3	2.8 (2.3-3.4)	108	53	**pHR HPV**
**0.5 (0.2-0.9)**	6	1.7 (1.3-2.1)	65	66	
**0 (0.0-0.0)**	0	0.3 (0.1-0.4)	10	26	
**0.1 (0.0-0.2)**	1	0.3 (0.2-0.5)	12	73	
**0.1 (0.0-0.2)**	1	0.3 (0.1-0.5)	12	82	
**0.1 (0.0-0.2)**	1	0.2 (0.1-0.3)	6	70	
**2.7 (1.8-3.6)**	34	5.6 (4.8-6.2)	214	16	**HR HPV**
**0.2 (0.0-0.6)**	3	0.9 (0.7-1.2)	35	18	
**0.2 (0.0-0.4)**	2	0.6 (0.4-0.8)	23	31	
**0.1 (0.0-0.2)**	1	0.5 (0.3-0.7)	19	35	
**0.2 (0.0-0.6)**	3	1.2 (0.8-1.5)	45	39	
**0.1 (0.0-0.2)**	1	0.6 (0.4-0.9)	24	45	
**0.9 (0.4-1.4)**	11	1.9 (1.5-2.3)	72	51	
**0.6 (0.2-1.1)**	8	2.2 (1.8-2.7)	86	52	
**0.4 (0.1-0.7)**	4	0.9 (0.6-1.2)	33	56	
**0.1 (0.0-0.3)**	1	0.7 (0.5-1.0)	28	58	
**0.1 (0.0-0.2)**	1	0.5 (0.3-0.8)	21	68	
**0.2 (0.0-0.6)**	3	0.3 (0.1-0.4)	10	59	
**0.1 (0.0-0.3)**	1	0.2 (0.1-0.3)	7	33	
**14.1 (12.4-15.7)**	174	42.1 (40.2-43.9)	1620	Mixed infection*	
**100.0**	1244	100.0	3841		**Total**

**Table 2:** The prevalence of HPV genotypes in mixed infections in Iranian female and male population

HPV genotypes	Females		Males	
More frequent genotypes with other HPV genotypes in mixed infections*	No	%, 95% CI	No	%, 95% CI
HPV-6	659	40.7 (38.2-43.0)	139	79.9 (73.6-85.6)
HPV-11	236	14.6 (13.0-16.5)	40	23.0 (16.7-29.3)
HPV-16	426	26.3 (24.3-28.6)	34	19.5 (13.8-25.9)
HPV-52	282	17.4 (15.6-19.2)	32	18.4 (12.7-24.1)
HPV-53	259	16.0 (14.2-17.8)	15	8.6 (4.6-13.2)
HPV-31	247	15.2 (13.5-17.1)	13	7.5 (4.0-12.1)
HPV-51	236	14.6 (13.0-16.5)	24	13.8 (9.2-19.0)
HPV-66	232	14.3 (12.7-16.1)	16	9.2 (5.2-13.8)
HPV-39	178	11.0 (9.4-12.5)	9	5.2 (2.3-8.6)
HPV-18	143	8.8 (7.3-10.3)	11	6.3 (2.9-10.3)
HPV-45	84	5.2 (4.2-6.4)	2	1.1 (0.0-2.9)
Total number of mixed infections	1620		174	

**Table 3:** The frequency and percentage of HPV infections of 31 Iranian provinces among HPV DNA samples

Province	Frequency	%*, 95% CI
Alborz	296	5.8 (5.2-6.5)
Ardabil	80	1.6 (1.2-1.9)
Bushehr	45	0.9 (0.6-1.1)
Chaharmahal and Bakhtiari	31	0.6 (0.4-0.8)
East Azerbaijan	68	1.3 (1.0-1.7)
Esfahan	72	1.4 (1.1-1.8)
Fars	67	1.3 (1.0-1.7)
Gilan	817	16.1 (15.0-17.1)
Golestan	23	0.5 (0.3-0.6)
Hamadan	11	0.2 (0.1-0.4)
Hormozgan	115	2.3 (1.9-2.7)
Ilam	2	0.0 (0.0-0.1)
Kerman	35	0.7 (0.5-0.9)
Kermanshah	61	1.2 (0.9-1.5)
Khuzestan	165	3.2 (2.8-3.8)
Kohgiluyeh and Boyer-Ahmad	5	0.1 (0.0-0.2)
Kurdistan	68	1.3 (1.0-1.7)
Lorestan	69	1.4 (1.0-1.7)
Markazi	139	2.7 (2.3-3.2)
Mazandaran	286	5.6 (5.0-6.3)
Northern Khorasan (Bojnord)	62	1.2 (0.9-1.5)
Qazvin	55	1.1 (0.8-1.4)
Qom	203	4.0 (3.5-4.6)
Razavi Khorasan (Mashhad)	43	0.8 (0.6-1.1)
Semnan	25	0.5 (0.3-0.7)
Sistan and Baluchestan (Zabol)	40	0.8 (0.6-1.0)
South Khorasan (Birjand)	11	0.2 (0.1-0.4)
Tehran	2073	40.8 (39.4-42.1)
West Azerbaijan	58	1.1 (0.9-1.5)
Yazd	48	0.9 (0.7-1.2)
Zanjan	12	0.2 (0.1-0.4)
Total	5085	100.0

Overall, the mixed HPV infections were found in 14.0% (n=174) of HPV DNA positive males, while 42.2% (n=1620) of HPV infection in females were caused by mixed HPV genotypes. Concomitant infection with at least two LR HPV genotypes was detected in 3.3% (168/5085) of all positive cases. In both sexes with mixed HPV genotypes, concurrent infection by at least one HR and one LR HPV genotype was commonly observed than other forms of mixed infections (Table 2). Only 0.3% (n=11) of females had pHR HPV genotypes as the mixed infection, and no similar cases were not found in males (Table 4).

**Figure 2:**
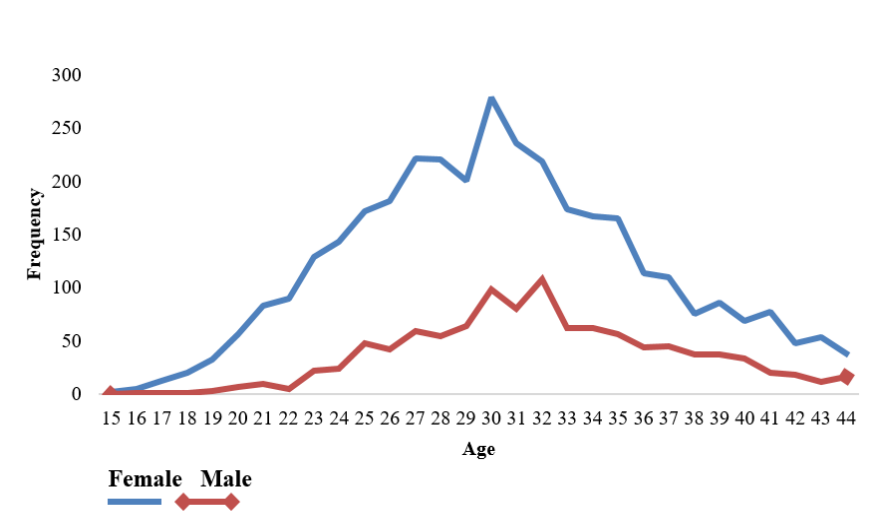
The frequency of HPV infection according to the age in the studied males and females

A total of 5,085 samples which were positive for HPV DNA were selected for more statistical analysis. They were divided into 4 age groups: 13-29, 30-44, 45-59, and 60-74 years (Table 5). In our study, like other studies (9, 13) no statistically significant correlation was found between HPV infection and age (*P*=1); however, 90.6% and 86.4% of the HPV infections in female and male subjects were seen in subjects aged 13 to 44 years, respectively. The highest rate of HPV infection occurred in positive subjects aged 30-44 years (Table 5). Figure (2) shows an upward trend of the frequency of HPV infection by age increasing with a peak at 30 and 32 years in both sexes. Along with age increasing, the rate of infection by HPV declines steadily and the lowest rate is seen in the age range 60-74 years. 

Distribution of HPV genotypes based on age groups was different in both sexes. In female subjects, HPV-16, as the most HR HPV genotypes, was more observed in the age group 30-44 years. The second and third common HPV genotypes were HPV-53 and HPV-52, which were detected more frequently in the age group 30-44 years. Interestingly, these three genotypes were not found in female subjects older than 60 years old. HPV-6, as commonly detected LR HPV genotypes, was almost equal in two age groups (13-29 and 30-45 years). In male subjects, the subjects aged 30-44 years had the first and second most frequent HR HPV and LR HPV genotypes: HR HPV-16, -51/-52 (the frequencies of HPV-52 and HPV-53 were equal), and LR HPV-6, -11. In the participants >60 years of age, HPV-6 and -11 were the most detectable HPV genotypes (data not shown). Table 6 shows HPV prevalence and three common genotypes of noncancerous cervical samples in Iran’s neighbouring countries.

**Table 4:** The prevalence of HPV genotypes in the mixed infections*

	Female		Male	
	No	%**, 95% CI	No	%**, 95% CI
LR HPV genotypes	139	3.6 (3.1-4.2)	29	2.3 (1.5-2.3)
HR or/and pHR HPV genotypes	487	12.7 (11.6-13.8)	16	1.3 (0.7-2.0)
HR and LR HPV genotypes	994	26.0 (24.5-27.2)	129	10.4 (8.7-12.2)
Total	1620	42.2 (40.6-43.7)	174	14.0 (12.1-15.8)

**Table 5:** Distribution of HPV genotypes by age groups and sex

HPV genotypes	Sex		13-29 y	30-44 y	45-59 y	60-74 y	**Total**
LR HPVs	F	No (%)*	630 (12.4)	738 (14.5)	142 (2.8)	11 (0.2)	
	M	No (%)	277 (5.4)	591 (11.6)	128 (2.5)	19 (0.4)	
HRor/and pHR HPVs	F	No (%)	493 (9.7)	715 (14.1)	100 (2.0)	16 (0.3)	
	M	No (%)	35 (0.7)	55 (1.1)	9 (0.2)	1 (0.0)	
HR and LR HPVs	F	No (%)	454 (8.9)	452 (8.9)	86 (1.7)	4 (0.1)	
	M	No (%)	35 (0.7)	82 (1.6)	11 (0.2)	1 (0.0)	
Total		No (%)	1924 (37.8)	2633 (51.8)	476 (9.4)	52 (1.0)	**5085 (100)**

**Table 6:** HPV prevalence and three common genotypes of noncancerous cervical samples in Iran’s neighbouring countries

Country	HPV prevalence in healthy female subjects** (Type of patients)	Three common genotypes	HPV genotyping assay	Reference
Turkey	17.9% (patients with normal pap smear)	HPV-16 (3.6%),	line blot assay	(30)
		HPV-6 (2.6%),		
		HPV-45 (2.2%)		
Pakistan **(Punjab)**	4.74% (patients with normal cervical cytology)	HPV-6 (25%),	the partial HPV L1 region sequencing	(31)
		HPV-55 (22.9%),		
		HPV-11 (20.8%)		
Qatar	7.6% (patients with normal and abnormal cervical cytology)	HPV-81 (34.5%),	real-time PCR and Sanger sequencing	(32)
		HPV-11 (31.0%),		
		HPV-35 (6.9%)		
Iraq (Kurdistan Region)	12.5% (Patients with vaginal discharge)	HPV-16 (53.8%)	Conventional PCR	(33)
Kuwait	2.4% (patients with normal cervical cytology)	HPV-11 (22.5%),	PCR-based sequencing	(34)
		HPV-81 (18.3%),		
		HPV-61 (12.6%),		
**The Kingdom of Bahrain**	9.8% (patients with normal pap smear)	HPV-52 (1.4%),	PCR DNA enzyme immunoassay (PCR-DEIA) and LiPA25 system	(35)
		HPV-16 (1.1%)		
		HPV-31 (1.1%)		
		HPV-51 (1.1%)		

## Discussion

HPV distribution varies in different countries and populations and even though in various parts of a country. For example, HPV-16 in Zhejiang province, HPV-52 in Guiyang and Guangdong provinces, and HPV-6 in Xi'an province of China are predominant genotypes (14, 15). In this study, we examined 10,266 Iranian male and female subjects with normal pap-smear, cell collections from vagina, cervix, and bulge of penile and anal sites of males from 31 provinces which showed an overall HPV prevalence of 49.5%. Based on a global study on female subjects with normal pap-smear, this rate of HPV prevalence was much higher than both neighboring countries and other regions in the world. The highest burden of HPV infection was estimated in African and Latin American countries and the lowest in southern Europe and South East Asia (12). No fixed pattern of the HPV genotypes distribution observed even in different parts of a province, e,g, the samples from suburb of Tehran commonly demonstrated mixed infections (the data were not shown). Although no sociodemographic data are available, low education and inappropriate economic conditions may be an explanation, high risk HPV distribution is affected by sexual behavior, host immune factors, environmental factors, smoking, having multiple sex partners, and the presence of other sexually transmitted infections (16). Therefore, HPV as an important transmitted infection causes some critical HPV-associated cancers in human, circulating HPV genotypes should be available to plan better the prevention. In different studies done on routine pap-smear samples in Iran, HPV-16 and -18 represented as the main genotypes (13, 17, 18), which are in disagreement with our results, as HPV-18 contained 9^th^ or higher in our study. Vaccinated female may reduce the HPV infections in male subjects, due to herd immunity (19); hence, domination of HPV-6, -11, -16, and -52 in female subjects of this study may explain the prevalence of these HPV genotypes in male subjects or vice versa. Table (6) displays the overall HPV genotypes prevalence and three frequent HPV genotypes in female subjects of Iran’s neighboring countries. Not only no common distribution pattern was observed between Iran and its neighbors, but also our findings are in contradiction with previous surveys conducted in Iran. In addition, in Saudi Arabia, as a country which Iranian people travel annually for pilgrimage, HPV-16, -18, and -11 were reported as three common genotypes (20). According to the latest report, cervical cancer is the 12^th^ most common cancer among Iranian female (7). On the one hand, based on our findings, the major genotype circulating in the normal population is HPV-6, which may justify the reduction of cervix cancer in the country. On the other hand, HPV-16 as the most important HR HPV genotype in the studied population and cancerous samples (3) has generally four lineages (A-D) that non-European variants (B-D) (21) play a key role in the development of an invasive form of cervical cancer (22). D is the prevalent lineage in Iranian females (23). Therefore, the importance of vaccination is well perceptible. In addition, there are no regular cervical cancer screening, either vaccination program or a national cancer registry. This study, in line with other previous studies, recommends to set up serious hygiene policies in case of HPV infections. 

Men with HPV infection only accounted for 12.1% of all HPV DNA cases. Among this, 86.0% had a single infection. The predominant three HPV genotypes found were in complete agreement with those were reported by Salehi-Vaziri in 2015 (9). Only almost one-seventh of male subjects were positive for mixed infections compared to the female subjectss where less than half of them were infected with mixed HPV genotypes. In Iran, nearly all boys are circumcised before the age of seven because of religious beliefs. Based on two meta-analysis studies, although no association was observed between HPV clearance and HPV acquisition of new infections, there was a significant reduction in genital HPV infection prevalence in male circumcision (OR=0.57) (24, 25), which may be a possible reason in the reduction of HPV infections or mixed infections in male subjects of this study. 

The age distribution of HPV infection in both sexes showed a mono summit graph with a peak age of 30-32 years. There was the highest incidence in the age of marriage and after beginning of the sexual activity due to some cultural habits, religious beliefs such as not to have illegitimate sex, increasing the age of marriage, and absence of immune protection against HPV in the lack of national vaccination program. Also, the rate of mixed infections is increased with the rise in the frequency of sexual contact and high-risk sexual behaviour (26). The steady decrease in the HPV infection rate in the middle age and elder individuals might explain the present immunity due to the natural history of HPV infection or self-limiting HPV infection within 18 months after infection as a result of host immune responses (15, 27). Although we had no information about the sexual activity of the participants or the status of HPV infection in parents of the children, self- or hetero-inoculation, the route of spreading of LR HPV infections in young people who have no sexual experiences, may justify the presence of HPV genotypes in the adolescents. (28, 29).

An important limitation of this study was the lack of cytology information of positive samples for correlation with the results of HPV genotypes. Another limitation, this rate of HPV prevalence may not reflect the distribution of HPV genotypes in the country since this study recruited only the samples with normal pap-smear, cell collections from vagina, and cervix in female subjects, and bulge of penile and anal sites in male subjects. Hence, similar studies need to be conducted on cancerous samples. No demographic data of participants such as marital status, job, and the number of sex partners were available. In spite of the male subjects’ limitations, this study gives an insight into the present status of the distribution of HPV genotypes in the target population and is helpful for setting policies.
